# The Human Body as a Super Network: Digital Methods to Analyze the Propagation of Aging

**DOI:** 10.3389/fnagi.2020.00136

**Published:** 2020-05-25

**Authors:** Harry J. Whitwell, Maria Giulia Bacalini, Oleg Blyuss, Shangbin Chen, Paolo Garagnani, Susan Yu Gordleeva, Sarika Jalan, Mikhail Ivanchenko, Oleg Kanakov, Valentina Kustikova, Ines P. Mariño, Iosif Meyerov, Ekkehard Ullner, Claudio Franceschi, Alexey Zaikin

**Affiliations:** ^1^Department of Chemical Engineering, Imperial College London, London, United Kingdom; ^2^IRCCS Istituto delle Scienze Neurologiche di Bologna, Bologna, Italy; ^3^School of Physics, Astronomy and Mathematics, University of Hertfordshire, Harfield, United Kingdom; ^4^Department of Paediatrics and Paediatric Infectious Diseases, Sechenov First Moscow State Medical University (Sechenov University), Moscow, Russia; ^5^Britton Chance Centre for Biomedical Photonics, Wuhan National Laboratory for Optoelectronics-Huazhong University of Science and Technology, Wuhan, China; ^6^Department of Experimental, Diagnostic and Specialty Medicine (DIMES), University of Bologna, Bologna, Italy; ^7^Laboratory of Systems Medicine of Healthy Aging, Lobachevsky State University of Nizhny Novgorod, Nizhny Novgorod, Russia; ^8^Complex Systems Laboratory, Discipline of Physics, Indian Institute of Technology Indore, Indore, India; ^9^Centre for Bio-Science and Bio-Medical Engineering, Indian Institute of Technology Indore, Indore, India; ^10^Institute of Information Technologies, Mathematics and Mechanics, Lobachevsky State University of Nizhny Novgorod, Nizhny Novgorod, Russia; ^11^Department of Biology and Geology, Physics and Inorganic Chemistry, Universidad Rey Juan Carlos, Madrid, Spain; ^12^Department of Physics (SUPA), Institute for Complex Systems and Mathematical Biology, University of Aberdeen, Aberdeen, United Kingdom; ^13^Department of Mathematics, Institute for Women’s Health, University College London, London, United Kingdom

**Keywords:** propagation of aging, network analysis, digital medicine, aging, inflammaging

## Abstract

Biological aging is a complex process involving multiple biological processes. These can be understood theoretically though considering them as individual networks—e.g., epigenetic networks, cell-cell networks (such as astroglial networks), and population genetics. Mathematical modeling allows the combination of such networks so that they may be studied in unison, to better understand how the so-called “seven pillars of aging” combine and to generate hypothesis for treating aging as a condition at relatively early biological ages. In this review, we consider how recent progression in mathematical modeling can be utilized to investigate aging, particularly in, but not exclusive to, the context of degenerative neuronal disease. We also consider how the latest techniques for generating biomarker models for disease prediction, such as longitudinal analysis and parenclitic analysis can be applied to as both biomarker platforms for aging, as well as to better understand the inescapable condition. This review is written by a highly diverse and multi-disciplinary team of scientists from across the globe and calls for greater collaboration between diverse fields of research.

## Introduction

Aging is the inescapable consequence of life that is common to all. However, the impact of aging on individuals can be very different, where some people live to a high age whilst maintaining excellent physical/mental health yet others may accumulate detrimental symptoms of aging relatively young. This leads to the distinction between “chronological” and “biological” age, where chronological age is an unwavering constant, biological age is a consequence of genetics, environmental exposure, and lifestyle and may be used as a metric to predict health risks. Unlike chronological aging, the rate of biological aging can change—the potential to distinguish biological from chronological aging, to treat or even reverse it, is the ambition of modern medicine.

**Figure 1:**
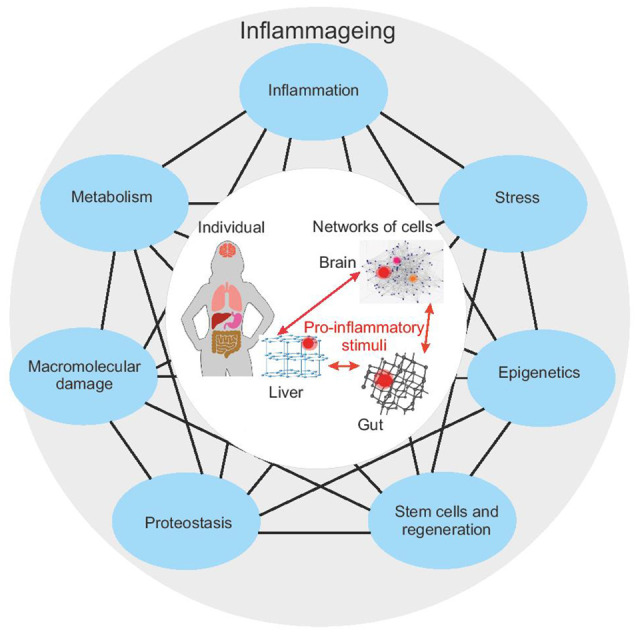
The seven pillars of aging according to Kennedy et al. (2014; blue outer nodes) are connected, such that perturbation in one pillar can affect each other pillar. The impairment of one or more pillars results in a chronic pro-inflammatory status—inflammaging. Inflammaging in a single part of the body can have distal effects on other systems (central circle) and thus can propagate aging. Studying the interaction of all these systems requires a systems biology approach. Given that each pillar or biological process can be represented by its network, this requires a network-of-networks solution.

Aging is a complex phenomenon in which the combination of genetic, environmental, and stochastic factors leads to highly personalized age-phenotypes. In the past years, researchers have attempted to identify the key tenants of the aging process (López-Otín et al., 2013; Kennedy et al., 2014; Figure 1). Despite some differences in the proposed hallmarks of aging, both the studies underline their large interconnectedness. These pillars are not discrete processes and an impact in any area can be propagated through all the other pillars.

Interestingly, the impairment of several (if not all) hallmarks/pillars of aging results in the accumulation of damaged and/or misplaced self-molecules that fuel inflammatory responses, promoting a status of chronic, low-grade and sterile (that is, occurring in the absence of infections) inflammation, that has been termed “inflamm-aging” (Franceschi et al., 2006). Although inflammaging is usually referred to as a systematic proinflammatory status, characterized by an increase in circulating levels of pro-inflammatory cytokines (such as IL-6, CRP), it should be taken into account that (1) inflammaging derives from a balance between pro-inflammatory and anti-inflammatory molecules; and (2) systemic inflammation is the result of the sum of multiple local inflammation events, occurring at the level of specific tissues, organs, and systems (Franceschi et al., 2017a). Inflammaging has been recognized as one of the main triggers of age-related diseases (Franceschi and Campisi, 2014; Furman et al., 2019). In turn, age-related diseases can promote a pro-inflammatory status, thus establishing a vicious circle between inflammaging and age-related diseases (Vitale et al., 2013; Franceschi et al., 2018).

To further compound the complexity, the mechanistic processes of aging occur on many levels, from the molecular (epigenetic, somatic mutation, metabolomic, etc.) in individual cells, tissues and organs, to the population level (genetic). At each strata, the rate and processes of aging and their contribution to inflammaging can be different, leading to a truly chimeric condition in both individuals and the greater population. Several studies suggest, for example, that the liver can age successfully, compared to other organs/systems (Bacalini et al., 2018; Morsiani et al., 2019), and yet this ability changes with age. Genome sequencing has demonstrated that cirrhotic livers have a higher mutational burden compared to normal livers (Brunner et al., 2019). As a consequence, the aging trajectories of an individual are the result of the interaction between organs/systems, each of which, in turn, derives from the combination of specific developmental programs, environmental exposures (i.e., biographies) and genetic backgrounds (Grignolio et al., 2014; Franceschi et al., 2017b, 2019).

Whilst it has been shown that analysis of serum metabolites can distinguish between different organ systems and nutrition (Sato et al., 2018), their variability and sensitivity to change make it challenging to correlate these to disease or age. However, the chemical environment of a cell is not only determined by its extracellular environment, but also by its genomic signature.

Several genomic loci have been associated with metabolites (reviewed in Suhre and Gieger, 2012) and a genome-wide association study, reported associations of 400 metabolites with 145 genomic loci (Shin et al., 2014). Analysis of single nucleotide polymorphisms (SNPs) associated with aging and longevity has identified genes in insulin-like growth factor signaling, DNA repair/telomerase maintenance and reactive oxygen species scavenging pathways. Dato et al. (2018) highlight that a single SNP can affect the resultant phenotype by its transmission through multiple genes, therefore to associate SNPs with aging requires the analysis of SNP-SNP interactions. Furthermore, we think this should be extended to links between SNPs and other genetic factors such as epigenetic marks. Analysis of the association between SNPs and aging underpins the genetic contribution to longevity. On one hand, this variation is relatively static and thus their analysis is not affected by chronological age or circadian rhythms. On the other hand, whilst SNPs can be associated with various phenotypic traits, variation in gene expression during aging as a consequence of environment (disease, diet, UV-exposure, etc.) is due to other regulatory pathways, such as epigenetic reprogramming.

Therefore, to fully understand aging, we should consider all age-related processes in unison. This is a challenging problem, not only from an experimental design perspective but also from the point of data analysis and mathematical modeling. Indeed, it is only now that such an approach may be conceivable using multi-‘omic technologies, big data analysis techniques, and super-computing. Significant advancement in the field of medical statistics has been made in the last decade with peak computing performance of 1EFLOP being achieved (Top500.org, 2020). With the corresponding technologies and frameworks, scientists and engineers have unprecedented opportunities to prototype and test various architectures of complex multilevel artificial neural networks and other deep learning techniques capable of analyzing such big data. Indeed, deep neural networks have been used to predict a person’s age using a basic blood test (Putin et al., 2017). In general, recent achievements of scientists show that modern systems, including those built on deep neural networks and supercomputer computations, open up new perspectives in the early diagnosis and treatment of several diseases.

This review, aimed towards researchers both in the field of biological aging as well as mathematical modeling, considers the use of multiplexed networks and longitudinal analysis to study the problem of aging, with examples drawn from the study of astroglial cell networks and epigenetics studies of the “biological clock.” We also consider approaches to study aging as a longitudinal, continuous phenomenon, rather than in a discrete-ordinal manner. Finally, we comment on the increasing complexity of this field, looking at the future directions, moving from population-level data to generating personalized aging profiles and treatments. The review calls for greater multi-disciplinary research to exploit modern and future capabilities for the study of aging and longevity.

## Epigenetic Aging and Biological Clock

Epigenetic modifications include a wide range of molecular mechanisms that play a pivotal role in the regulation of gene expression and genomic architecture. Among them, one of the best characterized is DNA methylation, a covalent modification of DNA that occurs preferentially at cytosines in a CpG dinucleotide. DNA methylation patterns are established early during development and can be stably maintained during cell divisions (Jones and Liang, 2009). Besides being relatively stable from a biological point of view, DNA methylation marks are well maintained during DNA and chromatin precipitation. This consideration, combined with the availability of several approaches to measure DNA methylation at a gene-targeted, genome-wide, and whole-genome level, makes this epigenetic modification an ideal candidate to identify longevity biomarkers. Indeed, DNA methylation is dynamically remodeled during several physiological and pathological conditions (Luo et al., 2018) including aging (Bacalini et al., 2017; Ciccarone et al., 2018; Unnikrishnan et al., 2019). Different types of changes to DNA methylation occurs during aging:

(1) Reproducible directional changes (prevalently hypermethylation, but also hypomethylation) of specific CpG sites (Hannum et al., 2013; Horvath, 2013);
(2) Hypomethylation of CpG sites within repetitive regions (Cardelli, 2018);
(3) Increase in the variability of methylation levels of a certain CpG position, considering a general population of individuals (Slieker et al., 2016);
(4) Increase in stochastic epi-mutations, that is, changes in DNA methylation levels of a certain CpG site that are not shared among the individuals of a general population.

So far, attention has been mainly focused on directional age-associated changes in DNA methylation and several CpG sites with tissue-specific age-dependent methylation levels have been described (Hannum et al., 2013). Unfortunately, it is often difficult to establish a causative link between DNA methylation remodeling and aging phenotype. In the studies that assessed methylation and gene expression from the same tissue, only a minor subset of genes with age-associated correlations between DNA methylation and gene expression was identified (Reynolds et al., 2014; Tserel et al., 2015). On the contrary, most of the loci showing hyper- or hypomethylation during aging were associated with genes with low transcription or without age-dependent expression changes (Reynolds et al., 2014; Tserel et al., 2015; Bacalini et al., 2018). Despite this, interesting hints resulted from the analysis of the pathways/ontologies enriched in loci with differential methylation during aging. Several of these studies were performed in whole blood or isolated blood cell types, and accordingly, pathways related to the regulation of immune functions were reproducibly enriched (Wang et al., 2016; Li et al., 2019). Other pathways enriched in loci with age-dependent methylation levels are linked to functions of the extracellular matrix (Wang et al., 2016; Li et al., 2017) and neurotransmission (Ong and Holbrook, 2014). Finally, it is worth noting that multiple studies reported that loci showing hyper- or hypomethylation with aging are enriched in bivalent chromatin domains, usually located in the promoters of developmentally regulated genes.

In recent years researchers have exploited the increased knowledge of age-associated directional changes by developing epigenetic clocks, which are mathematical models that combine the methylation of specific CpG sites (usually below 600) to provide an estimate of the epigenetic age of an individual (Bartlett et al., 2014). Several epigenetic clocks, differing in both the included CpG sites and the human tissues on which they have been validated, have been proposed (Hannum et al., 2013; Horvath, 2013; Weidner et al., 2014; Horvath et al., 2018; Levine et al., 2018). Although with some differences, these clocks have been comprehensively shown to detect age acceleration effects associated to different age-related conditions, spanning from neurodegenerative diseases to cancer and also prospectively reviewed in (Field et al., 2018; Horvath and Raj, 2018). Despite these successful results, much has still to be done in this sense. In particular, the use of appropriate mathematical approaches will likely permit us to develop epigenetic clocks based not only on directional changes in DNA methylation, but also on the other aspects of age-related DNA methylation remodeling (hypomethylation of repetitive elements, increase in variability and epimutations), thus improving the performance of predictors and broadening the spectra of age-related diseases that could benefit from early diagnosis.

## Network Modeling

Mathematical modeling aims to reduce complex problems into defined parameters; adjusting the parameters of the models to provide insight into real systems. Networks present a simple framework to model complex systems that comprise of a large number of interacting elements. The network for any biological system can be represented by nodes (vertices) and links (edges). For example, biomolecules may be represented by vertices and their intermolecular interactions by edges. In this way, all biological systems can be studied in a single framework. Network spectra (eigenvalues) are known to provide rich information on the topological structure and diffusion of signals within them (Sarkar and Jalan, 2018), providing an indirect blueprint of complex systems. With age-related diseases, cancer has received the most attention, from a network theory perspective. For example, network spectra provide a comprehensive approach to analyzing proteomic data for breast, oral, ovarian, cervical, lung, colon, and prostate cancer (Rai et al., 2017). This analysis demonstrated that the protein-protein interaction networks of the normal and cancerous tissues associated with the seven cancers have overall similar topological and spectral properties but some changes in the complexity were unique to different cancers under their study. Similarly, network spectra have been successfully used in many other instances to classify disease states from healthy states of a tissue (Jalan et al., 2015; Rai et al., 2015). Importantly, analysis of common proteins in all cancer networks have helped to reveal proteins which not only occupied significant positions in all the layers, but are also directly involved in causing cancer (Rai et al., 2017). The prediction and analysis of micro-RNAs targeting these proteins provide a hint towards their possible roles in tumorigenesis. This novel approach of network spectra should help in understanding cancer at the fundamental level and provide a clue to develop promising single-drug therapy for multiple diseases as well as personalized medicine.

Biological age acceleration, expressed in epigenetic biomarkers, has not been explicitly related to network signatures of cancer or other age-related diseases. The first step to address this was made by Krivonosov et al. (2020), where parenclitic network analysis (Zanin et al., 2014) was employed to characterize differential DNA methylation of mothers and siblings of Down Syndrome patients. Network indices revealed age and group dependence, and the constructed networks as a whole suggested some associated molecular functions, according to Gene Ontology analysis. The developed approach is a promising tool to access the other cases of accelerated and decelerated aging.

Simplifications are necessary and unavoidable to build a meaningful mathematical model to identify the major biological mechanisms. Finding the right balance between a detailed description and a deeper understanding is an enduring challenge. Already very strong simplifications can lead to unexpected and barely understood behavior as soon as large networks of interacting players are involved. The mammalian brain with its network of spiking neurons is probably one of the most prominent examples in biology. The individual neurons and the synaptic communication amongst them are quite well understood but the orchestrated function as a whole is still puzzling. Mathematical modeling offers an approach to bridge gaps in understanding. For example, at rest, the neurons in the brain are far from being inactive but generate spontaneous firing activity. Detailed functional magnetic resonance imaging (fMRI) of the spontaneous activity has been used as a baseline to classify task-related activation in cognitive studies. These have shown that resting-state activity, first considered as simple noise, contains much more structure and information in a complex non-Gaussian activity pattern, than previously the information contained can be used to reveal functional connections (DeWeese and Zador, 2006; Murphy et al., 2009; Harris and Thiele, 2011; Foster et al., 2016). Invasive and non-invasive electrophysiological recordings and fMRI reveal a remarkable correspondence between spontaneous and task-based parcellations of large-scale functional brain networks across many spatiotemporal scales. This demonstrates that structural properties of neural networks and their functional repertoire can be inferred by the spontaneous neural activity, with clinical applications (Fox and Greicius, 2010). However, the use of fMRI and its variants (e.g., time-varying functional connectivity fMRI) for studying neuronal connectivity remains somewhat controversial owing to the difficulty in suitable controls and a need for better statistical models (Lurie et al., 2020).

Substantial effort has been made to develop simple models of excitatory and inhibitory spiking neurons, aiming to mimic the cortical activity (Gutkin and Ermentrout, 1998; Rauch et al., 2003; Jolivet et al., 2004, 2006; Shlizerman and Holmes, 2012). A review by Gerstner and Kistler (2002) gives some guidelines for extracting relevant dynamical features of networks of integrate-and-fire neuron models to connect these with real measurements (Gerstner and Kistler, 2002). The spontaneous activity or the persistent, selective delay activity are examples of *in vivo* neuron properties that can be linked to simple integrate-and-fire neuron models.

Collective Irregular Dynamics (CID) in so-called balanced networks of spiking neurons can act as a mathematical testbed for the background activity at rest. Balanced networks are such that the excitatory and inhibitory activities compensate each other (Vogels et al., 2005). The CID is a dynamic phenomenon known from dynamic system theory and we propose to transfer the concept to spontaneous background activities observed in the brain. It is a macroscopically observable phenomenon that originated with an orchestrated interplay of individual neurons (Ullner et al., 2018). The considered neuronal networks of spiking neurons are random (Brunel, 2000; Ostojic, 2014). The network is free of any external driving or input and so the resulting complex behavior is fully self-generated. Although the setup of the mathematical model seems simple, the joint activity is far from being trivial. The overall scenario of CID in the balanced spiking network is reminiscent of the background activity of the brain at rest state.

How can such a paradigmatic model help the medicine to achieve healthy aging? The brain represents one of the target organs of damage for several diseases and undergoes structural and functional changes over its life span. For instance, classical galactosemia is a rare genetic metabolic disorder that impairs the ability to metabolize the sugar galactose. It results in chronic deterioration with a significant influence on the quality of life and general cognitive performance, including alterations to rest-state behavior (van Erven et al., 2017). In another recent example, fMRI or echocardiogram measurements pointed to a possible connection between the modulations of intrinsic resting-state and chronic migraines of female patients (Androulakis et al., 2017). These results demonstrated an overall decrease in resting-state functional connectivity of the default mode network, the salience network, and the central executive network in women with chronic migraines. The connections between the CID phenomenon, the brain’s background activity at rest, and age-related diseases are a speculative proposal at an early stage to illustrate the benefits and challenges of such a cross-disciplinary approach. However, mathematical models bridge gaps in knowledge and can be used as a hypothesis testbed to address critical conditions. Brain dynamics at rest might reveal early precursors before changes on cellular or organ levels are detectable. These findings, in turn, inform molecular biology to identify the underlying molecular mechanisms or to understand malfunctions on tissue or organ strata. The real benefit of mathematical modeling unfurls if neuronal data related to diseases are available. The mathematical model could be used to identify the critical network parameter that generates a malfunction. The continuous path from the healthy rest state to pathological behavior in the mathematical model might reveal early precursors to intervene the progression in patients.

Mathematical modeling is a powerful bottom-up tool if developed in close interaction with the biological and medical progress. Aging, understood as a dynamic system, has the potential to change the paradigm from a vain endeavor to fight a disease to a journey in a complicated and diversified landscape with many possible tracks (Hedden and Gabrieli, 2004; Grady, 2012).

### Applications to Age-Related Diseases

**Figure 2:**
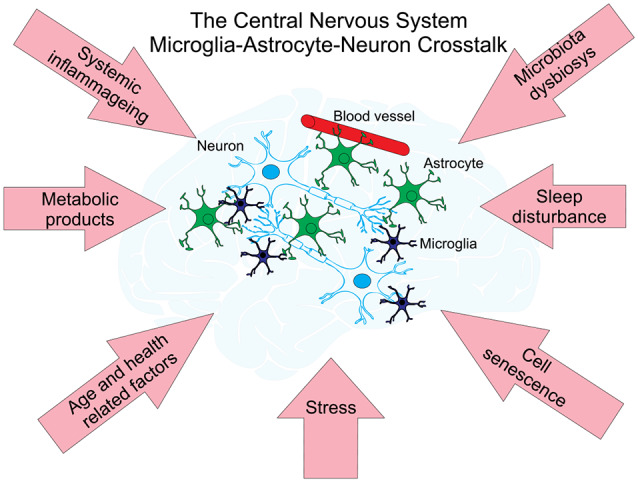
Neuronal networks—cell types and factors interacting with each other affect age-related diseases. There are three main types of cells within neural cellular networks. A neuron or nerve cell is an electrically excitable cell that communicates with other neurons *via* specialized connections called synapses. They are the basic (functional and structural) unit of nervous tissue and the central nervous system (CNS). Astrocytes support neuronal function by providing essential structural and nutritional support, neurotransmitter trafficking and recycling and may also contribute to brain information processing. Astrocytes function as versatile metabolic sensors of CNS milieu and play an important role in the maintenance of brain metabolic homeostasis (for a recent review see Marina et al., 2018). Microglia are the only immune cells that permanently reside in the CNS. In the past decade, studies on microglia have expanded from investigating their function as resident macrophages of the brain and mediators of injury, neuroinflammation and neurodegeneration (reviewed in Salter and Stevens, 2017; Tay et al., 2017) to understanding their origins and non-immunological roles in the CNS. Networks of these cells are under the influence of different factors affecting the development of age-related diseases (pink arrows) and are supporting their metabolism by the interchange of metabolic products with blood vessels.

Aging of the brain is associated with neurodegenerative disorders, the most prevalent of which is Alzheimer’s and Parkinson’s diseases. These are the most common causes of dementia in the elderly, affecting over 10% of the population over the age of 65 in the United States (Querfurth and LaFerla, 2010). Despite significant research progress, the pathogenesis of Alzheimer’s and Parkinson’s diseases remain fragmentarily understood, partly due to the extremely complex intercellular cross-talks taking place throughout the aging process (Henstridge and Spires-Jones, 2018; Jagust, 2018; Styr and Slutsky, 2018). Considering the complexity of cellular and molecular interactions, mathematical modeling provides a unique opportunity to further understand the pathogenetic mechanisms of age-related neurodegenerative disorders. There are two recent reviews about mathematical modeling efforts on the whole in neurodegenerative diseases (Lloret-Villas et al., 2017) and in particular in Parkinson’s disease (Bakshi et al., 2019). Noteworthy are several mathematical models of the pathogenesis of Alzheimer’s disease (AD) which describe the dynamic cross-talks that occur among microglia, astroglia, neurons, and amyloid-β (Aβ; Figure 2). Kyrtsos and Baras (2015) proposed a model to study the role of the glymphatic system induced clearance of Aβ from the brain via the perivascular space surrounding cerebral blood vessels in AD.

Experiments have shown that astrocytes play an important role not only in the process of elimination of soluble proteins and metabolites from the central nervous system (CNS; Rasmussen et al., 2018) but also in regulating cellular functions and information transmission in the nervous system (Perea and Araque, 2010; Araque et al., 2014). In contrast with neuronal cells, astrocytes do not generate electrical excitations (action potentials). However, their intracellular dynamics have shown similar excitable properties for changes in calcium concentration (Nadkarni and Jung, 2003; Semyanov, 2019). These signals can affect neuronal excitability and the efficiency of synaptic transmission between neurons by Ca^2+^-dependent release of gliotransmitters (e.g., glutamate, D-serine, ATP; Savtchouk and Volterra, 2018). It has emerged that astrocytes are interconnected into networks by gap junction channels. Networks of astrocytes accompanying neuronal cells generate collective activity patterns that can regulate neuronal signaling by facilitating or by suppressing synaptic transmission (Perea and Araque, 2010; Araque et al., 2014; De Pittà et al., 2016).

Despite efforts in recent years to model the role of astrocytes in information processing in the CNS (Oschmann et al., 2018; Kanakov et al., 2019), only a few computational models are investigating the role of astrocytes in neurodegenerative diseases. The most popular pathological behavior of astrocytes investigated by modeling is epilepsy (Ullah et al., 2009; Volman et al., 2012; Amiri et al., 2013; Tewari and Parpura, 2013) and AD (Lenk et al., 2016). These computational studies describe the abnormal astrocyte regulation of synaptic transmission and pathological release of gliotransmitters from astrocytes. There has been no model developed to study the age-induced changes in the morphology of astrocytes. However, in experimental studies, it was shown that pathology astrocytes undergo morphological and functional remodeling that is dependent on an injury, neurodegenerative disease, and aging processes (Dossi et al., 2018; Verkhratsky, 2019; Verkhratsky et al., 2019). Such models can be developed based on existing models that take into account realistic cell morphology (Savtchenko et al., 2018; Gordleeva et al., 2019; Wu et al., 2019). The role of astrocytes in neurodegenerative diseases and the aging process requires further investigation. Biophysical models of astrocytic regulation of synaptic transmission in neuronal circuits both at the level of individual cells and at the network level should be developed and investigated for aging based on experimental data. Simulation experiments in large-scale neuron-glial networks reproducing the signaling observed in experiments with aging and neurodegenerative diseases are expected. The need for such studies is related to the identification of targets for the effects of pharmacological agents in the treatment of neurodegenerative diseases caused by violations of neuronal signaling.

Further, there has been no model developed to investigate the role of cellular senescence and the propagation of senescent associated secretory phenotype (SASP) molecules through brain tissue in aging and age-related diseases (Baker and Petersen, 2018). This idea has been conceptualized as inflammageing linked to garb-aging (Franceschi et al., 2018) and is based on a hypothesis that the progressive accumulation of senescent cells (and their pro-inflammatory SASP phenotype) in all organs and tissues contribute to aging/inflammaging and this state can propagate through the tissue or brain network. It will be very interesting to develop a network model describing the interaction between healthy and senescent microglia and astrocytes, the concentration of garbage accumulated during neuronal activity (cellular and molecular garbage: cell debris, resulting from cell death, misplaced/altered/oxidized molecules, gut microbiota products, internal exposome, among others) and cleaned by healthy glial cells via the glymphatic system (Benveniste et al., 2019), and propagation of the signaling SASP molecules in some volume of the brain tissue. Models of such type can help to understand the mechanisms of the inflammageing propagation through the brain network resulting in aging and age-related diseases.

On a molecular level, computational modeling could be a useful way to study AD by handling numerous parameters related to ion channels and electrophysiology. We have noted 10 models are published in ModelDB with the software NEURON (Markaki et al., 2005; Ferrante et al., 2008; Morse et al., 2010; Bhattacharya et al., 2011; Culmone and Migliore, 2012; Romani et al., 2013; Bianchi et al., 2014; Rowan et al., 2014; Coskren et al., 2015; Rumbell et al., 2016; Table 1). Here, neural networks are introduced and interfaced with amyloid effect and chemical or electrical stimulation. So far, different channels, chemical agents, synapses, and morphological properties have been modeled for AD. As we understand more about the mechanisms modulating the excitability of AD neurons to a greater extend, modeling brings insights into how to mediate the ongoing damage of AD by chemicals or low-intensity electrostimulation. However, comprehensive modeling of the neural environment, e.g., the role of glial cell-networks during AD is missing.

**Table 1:** Summary of 10 computational models on AD study.

Model type	Cell type	Working mechanisms	Main results	Reference
Multi-compartment	CA1 pyramidal neuron	Incorporate different calcium channels	Decreased excitability of aged CA1 cells	Markaki et al. (2005)
Multi-compartment	CA1 pyramidal neuron	Endogenous and exogenous chemical modulation on membrane	Explore the effect of chemicals on neural diseases	Ferrante et al. (2008)
Multi-compartment	CA1 pyramidal neuron	Blocking A-type K^+^ currents	Back-propagating action potentials in the dendrites induce hyperexcitability	Morse et al. (2010)
Neural network	Non-specific cells in thalamus and cortex	Excitatory and inhibitory connectivity	Active synapses in the thalamus decrease alpha-band EEG wave	Bhattacharya et al. (2011)
Multi-compartment	CA1 pyramidal neuron	Aβ-peptides progressive accumulation	Multi mechanisms modulate excitability	Culmone and Migliore (2012)
Single neuron with synapses input	CA1 pyramidal neuron	Aβ-induced enhancement of release probability	Alter the spike probability of CA1 pyramidal neurons	Romani et al. (2013)
CA1 network	CA1 pyramidal neurons with interneurons	Increasing the cAMP Response Element Binding protein	CREB-based therapies for AD	Bianchi et al. (2014)
Neural network	Cell populations in one column	Low-intensity electrostimulation	Raise activity and break ongoing damage	Rowan et al. (2014)
Multi-compartment	Pyramidal neuron in monkey cortex	Combining morphology and ion channels	Membrane resistance and changed morphology affect excitability	Coskren et al. (2015)
Multi-compartment	Pyramidal neuron in monkey cortex	Automated parameter optimization	Get many parameters fitting the model	Rumbell et al. (2016)

*Abbreviations in the table: Electroencephalogram (EEG); cAMP Response Element Binding Protein (CREB); Alzheimer’s disease (AD)*.

In addition to the amyloid hypothesis, brain inflammation (increased microglia and astrocyte activation) has been increasingly recognized as a potential mechanism of AD pathogenesis (Heppner et al., 2015; Parbo et al., 2017; Sawikr et al., 2017). Evident changes have been found in microglia and astroglia in the post-mortem brains of AD patients (Heneka et al., 2015). Also, genome-wide analysis suggests that several genes increasing the risk of AD modulate the glial clearance of misfolded proteins and inflammation. The understanding of immune/inflammatory pathways in AD and their regulatory mechanisms should offer opportunities for drug development targeting neuroinflammation (Fu et al., 2019). However, to date, most of the anti-inflammatory drug candidates undergoing clinical trials have failed. Thus, a systems approach to studying AD by combining detailed morphological reconstruction and advanced neural network modeling to cover both neurons and glia of the AD brain may highlight new therapeutic opportunities. The quantitative and systems thinking will provide a big picture for probing AD and effective treatment approaches in the future.

## Emerging Strategies for Early Diagnosis of Age-Related Diseases: Bayesian Estimation, Neural Networks and Parenclitic Analysis

It has been shown that many of the molecular and cellular mechanisms involved in aging are closely related to those driving the appearance and development of cancerous tumors, either because they are shared or because they are divergent (Finkel et al., 2007; Aunan et al., 2017). Such mechanisms include the role of genomic instability, telomere attrition, epigenetic changes, loss of proteostasis, decreased nutrient sensing and altered metabolism, cellular senescence, and stem cell function (Maslov and Vijg, 2009; Campisi, 2013; Hou et al., 2015). As a consequence, the recent exploration and progress of new technologies to detect early signs of oncological disorders should also be relevant for the assessment of significant mismatches between chronological and biological age.

One of the main trends of modern healthcare is directed towards personalized medicine. All individuals differ in genotype and phenotype and thus should be managed differently for disease prevention, detection, and treatment. Modern ‘omics technologies are capable of acquiring large amounts of quantitative, or semi-quantitative data (mass spectrometry, quantitative PCR, microarrays, etc.) relatively cheaply, thus there is a large potential to delivering truly personalized medicine based on an individual’s molecular profile.

Significant improvements in screening procedures for early cancer detection can be attained by using quantitative tools for the analysis of longitudinal biomarkers—instead of simple cut-off values (McIntosh et al., 2002). This has been recently shown, e.g., for the case of invasive epithelial ovarian cancer, where the use of a single threshold rule is the current norm for interpretation of serum Cancer Antigen 125 (CA125) as a first-line test in ovarian screening (Blyuss et al., 2018). It was demonstrated in the recent United Kingdom Collaborative Trial of Ovarian Cancer Screening (UKCTOCS; Menon et al., 2015), that it is not an individuals’ CA125 measurement that indicates cancer development, rather a deviation from personal baseline. Therefore, recent approaches in ovarian cancer are directed towards constructing personalized baselines based on patients’ serial measurements with analyzing further sequential measurements from the perspective of previous history (Whitwell et al., 2020). For example, three approaches have been applied to longitudinal serological data from ovarian cancer: (1) the methods of mean trends (MMT) algorithm (Blyuss et al., 2018) which evaluates the dynamics of longitudinal markers using weighted derivatives of marker changes as well as the average area under the time series, coefficient of variation and “center of mass” as predictors in logistic regression; (2) The Risk of Ovarian Cancer Algorithm (ROCA), that fits Bayesian hierarchical change-point model on CA125 serial data (Skates et al., 2001); and (3) Parametric Empirical Bayes (PEB) that evaluates deviation from normality based on population characteristics such as the population mean and within-subject and between-subject variances. They significantly outperform single CA125 cut-offs, demonstrating the effectiveness of the personalized approach, both in terms of area under the receiver operating curve (AUC) and in terms of sensitivity at a fixed, clinically relevant, specificity.

Sophisticated procedures for early detection of oncological diseases, such as Bayesian computation methods or deep learning techniques (Goodfellow et al., 2016), involving more than one biomarker can further reduce human intervention in the diagnostic process. In particular, it has been recently shown (Mariño et al., 2017) that the combined analysis of a group of specific biomarkers (namely CA125 and Human Epididymis Protein 4 or Glycodelin) improves the detection of change-points (from personal baseline to deviance) in multiple time series data (compared to the analysis of CA125 alone) which, in turn, can be associated with the development of tumors. Similar processes related to the loss of proteostasis play a key role in biological aging and they can be detected at an early stage employing the same class of quantitative analysis techniques.

Although not as straightforward to interpret, from a clinical point of view, as Bayesian models, deep learning techniques are currently attracting attention in many biomedical applications. In particular, recurrent neural networks can integrate information of multiple biomarkers without the need to construct explicit probabilistic models, as opposed to Bayesian analysis methods. This has been recently shown in (Vázquez et al., 2018), where a quantitative performance study of these two approaches for the diagnosis of ovarian cancer from longitudinal biomarker data has been carried out.

The challenge with large, multi-omic data sets is in analyzing them in a biologically meaningful manner. The difficulty in interpreting large scale data sets is due to the non-linearity of molecular pathways; i.e., for each pathway, there are multiple branching points and multiple levels of regulation such that a perturbation of a single analyte (mRNA, protein, metabolite) may have a cellular effect that is not immediately obvious (Haas et al., 2017). Therefore, taking large data sets and analyzing fold-change of single analytes (mRNA, protein, metabolites, etc.) without taking into account everything else, lacks biological context. To overcome this, it is possible to use pre-defined annotations (gene ontology, pathways) to identify biological patterns. However, this requires prior knowledge regarding the analytes function, currently (November 2019) Swissprot, a manually curated database of proteins contains 561, 356 annotated proteins, whereas TrEMBL, a related database comprised of computationally curated annotations contains 181, 787, 788 proteins—highlighting the enormous black hole that exists in experimentally verified annotations (Bateman et al., 2017), a crucial limitation in these approaches.

One way to address this issue is to use techniques that require no *a priori* knowledge of the analytes. Parenclitic networks, first published by Zanin et al. (2014), identify global changes between two data sets through graph-based analysis, where nodes (vertices) represent analytes and edges between nodes are present if that pair of analytes differ between the two data sets. Thus, analytes that are changing become well connected within the network whereas those that change only a little or not at all are weakly connected. Since the construction of these networks is not based on fold change or *p*-value (α-value) they are not affected by the inherent bias of these commonly used statistics. The seminal parenclitic article analyzed transcript data from Arabidopsis, and since then have been applied to DNA methylation data (Karsakov et al., 2017), proteomic data (Whitwell et al., 2018) and credit card fraud detection (Zanin et al., 2018). A full description of how to construct parenclitic networks are presented in Whitwell et al. (2018), in which multiple approaches to network construction and the integration of categorical variables into the network are also discussed. When applied to ovarian cancer, the networks provide two levels of information. First logistic regression models of the network topologies were able to distinguish case/control, and second, analysis of individual nodes suggested granzyme H and fibroblast growth factor-binding protein 1 as changing as early as 34 months pre-diagnosis.

The critical feature of longitudinal analysis is to detect, in an individual, when a marker is changing, thus overcoming natural variation of an analyte within a population that may mask diagnosis using simple threshold-based diagnosis. Of course, whilst it is trivial to include new biomarkers in logistic regression models, the lack of available biomarkers hampers the application of this approach. Combining longitudinal analysis with holistic techniques, such as parenclitic networks, that can exploit personalized ’omics screening (e.g., routine transcriptomic, proteomic analysis) could be an important advancement in this field.

## Summary and Future Perspective

Without any doubt, the rapid development of artificial intelligence will lead to a new generation of personalized patient tools. Each patient will be associated with a digital profile, analyzed by an algorithm, which will recommend a personalized treatment based on previous learning, data mining, and even communication with other artificial intelligence algorithms through the worldwide web.

It remains that the practical utilization of neural networks for the analysis of medical data is a challenging problem. Recently there has been explosive-like progress in the development of artificial intelligence machine learning methods for pattern recognition of different kinds. These results have included deep learning convolutional neural networks, generative adversarial networks, and state-of-the-art architectures of recurrent neural networks including Long Short Time Memory and Gated Recurrent Unit networks. However, in contrast to image processing, except for some rare examples (Angermueller et al., 2017; Putin et al., 2017), the application of deep learning neural network for the early diagnosis of cancer (and thus applications for aging), based on the analysis of proteomic and epigenetic data has not progressed a lot. The major challenge is the application of deep neural networks for an analysis of high dimension low sample size data vital for diagnostics of age-related diseases. Feature selection methods, such as Lasso, have been suggested to solve this problem. However, Lasso ignored the nonlinearity and interactions among features. More efficient methods have included Hilbert-Schmidt Independence Criterion Lasso (HSIC-Lasso) and Least Angle Nonlinear Distributed feature selection (LAND) methods (Yamada et al., 2016), and did not require training with a large sample size. The same advantage was implemented in the Deep Neural Pursuit networks (Liu et al., 2017) and deep feature selection. The efficiency and usability of these new methodologies seem to be very promising and are under active investigation now. This approach should be also definitely linked with other network methods because network biology can provide insightful models for genetic phenomena such as penetrance, epistasis, and modes of inheritance, all of which are integral aspects of Mendelian and complex diseases (Furlong, 2013). In particular, it looks very promising to link deep learning networks with recently developed parenclitic network analysis (Whitwell et al., 2018). The advantage of this approach is the possibility to represent data in the form of a connected graph, even in the cases when no known interactions between parameters are available. The outcomes of this representation can be then used for training the deep neural network. This approach will, however, require a detailed investigation of this methodology and comparison with other abovementioned methods and with well-established machine learning algorithms such as feature vector machines, random forest, or other sparse methods.

Effective data analysis will be impossible without an understanding of underlying biological mechanisms and, hence, we should work on the integration of data analysis and mathematical modeling. The most challenging problem here is the automatic integration of experimental data with mathematical models. Many fundamental principles governing brain functioning are unclear: What are its properties? How do these properties change over time? How to integrate realistic morphological data into computational modeling of aging-related neural diseases?

Through this review, we have highlighted and discussed several analytical tools and modeling approaches that can be applied to the field of personalized medicine and aging. The very realistic future of personalized medicine and understanding of the complex biological super-network underpinning aging lies in the conflation of these ideas.

## Author Contributions

All authors listed have made a substantial, direct and intellectual contribution to the work, and approved it for publication. CF established this research collaboration. CF, AZ, SG, MI and HW conceived the manuscript. All authors contributed to writing the manuscript. SG created the figures. HW compiled and edited the manuscript.

## Conflict of Interest

The authors declare that the research was conducted in the absence of any commercial or financial relationships that could be construed as a potential conflict of interest.
